# Thrombosed traumatic aneurysm of the occipital artery: a case report and review of the literature

**DOI:** 10.1186/1752-1947-6-203

**Published:** 2012-07-17

**Authors:** Vikas Y Rao, Steven W Hwang, Adekunle M Adesina, Andrew Jea

**Affiliations:** 1 Division of Pediatric Neurosurgery, Texas Children’s Hospital, Department of Neurosurgery, Baylor College of Medicine, Houston, TX, USA; 2 Division of Pediatric Neurosurgery, Department of Neurosurgery, Floating Hospital for Children, Tufts Medical Center, Boston, MA, USA; 3 Division of Neuropathology, Texas Children’s Hospital, Department of Pathology, Baylor College of Medicine, Houston, TX, USA

## Abstract

**Introduction:**

Occipital artery aneurysms are very rare vascular lesions. Most cases reported in the literature have been post-traumatic pseudoaneurysms of the occipital artery.

**Case presentation:**

We report the case of a 14-year-old Caucasian boy presented with a painless non-pulsatile scalp mass that developed rapidly after minor blunt head trauma. The scalp mass was excised six months after the trauma. A pathologic diagnosis of a thrombosed true aneurysm was made. Our patient has had no recurrence of the mass at 15 months follow-up.

**Conclusions:**

We present a case of a true aneurysm of the occipital artery following minor head trauma. We review the literature for similar cases and discuss the difficulty of establishing a diagnosis prior to surgical intervention.

## Introduction

Aneurysms of the external carotid circulation are rare. Of these aneurysms, scalp aneurysms involving the occipital artery are the rarest. They have been described primarily as a consequence of blunt, penetrating or iatrogenic trauma. There are also cases secondary to infection and autoimmune disease, and cases of spontaneous aneurysms of idiopathic etiology.

Pathologic examination of these lesions reveals that the majority are pseudoaneurysms, the dilated walls of which are comprised of only the outer layers of the native vessel wall. These lesions usually present as pulsatile scalp masses and are often painless.

Only 10 cases of traumatic and spontaneous aneurysm of the occipital artery have been previously reported in the English literature, with sporadic cases in other languages. Of these previously reported cases, only one was confirmed to be a true aneurysm of the occipital artery, comprising all layers of the arterial wall. We report another case of a true aneurysm of the occipital artery in a pediatric patient.

## Case presentation

A 14-year-old Caucasian boy presented to our Pediatric Neurosurgery clinic for evaluation of a scalp mass. Our patient was a previously healthy child who had a basketball strike to the back of his head four months prior to presentation. Our patient did not have loss of consciousness and had immediate scalp swelling near the impact site. While initially painful, the pain subsided over the next two days, but a firm mass persisted for months after the incident. He was then sent to our Pediatric Neurosurgery clinic for evaluation of the persistent mass.

**Figure 1:**
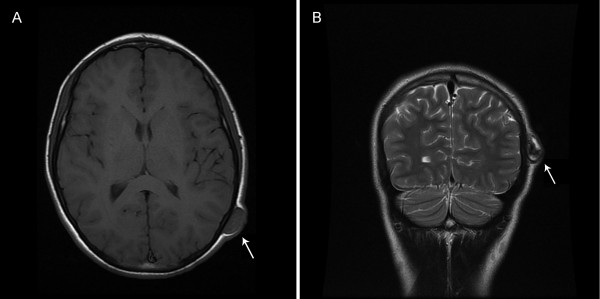
**Preoperative T1-weighted axial and T2-weighted coronal magnetic resonance imaging.** (**A**) Preoperative T1-weighted axial magnetic resonance imaging and (**B**) T2-weighted coronal magnetic resonance imaging shows an evolving hematoma (arrow) in the vicinity of the occipital artery.

On examination, our patient was found to have a 2.5cm firm, non-tender, non-pulsatile, mobile mass in the left posterior temporal area approximately 6.5cm superior to the mastoid tip and 4.5cm lateral to the external occipital protuberance. The mass was not bothersome to our patient other than it being unsightly. Upon palpation, no palpable thrill was present, and we did not auscultate the lesion. A skull X-ray showed no bony abnormalities. A magnetic resonance imaging scan showing heterogeneity within the lesion suggested partial thrombosis of the scalp mass or evolving hematoma of differing ages (Figure 1). Due to the very mild symptoms, the decision was made to observe the lesion. Two months later, however, our patient returned with cosmetic concerns related to the mass. Surgery was offered to remove the lesion and provide a definitive diagnosis.

Our patient was brought to the operating room, and a curvilinear skin incision was made superior to the mass. The firm, smooth, non-pulsatile mass was encountered just under the galea aponeurotica. It was circumferentially dissected and associated with brisk arterial bleeding from vessels proximal and distal to the lesion. We had not anticipated an aneurysm and, therefore, encountered significant bleeding until the proximal and distal arteries were coagulated. The bleeding was easily controlled with bipolar electrocautery and division of the proximal and distal artery. The mass was excised en bloc and sent for pathologic examination. However, we did not section the mass on the operating table to look for intraluminal thrombosis. Our patient tolerated the procedure well and was sent home later the same day.

**Figure 2:**
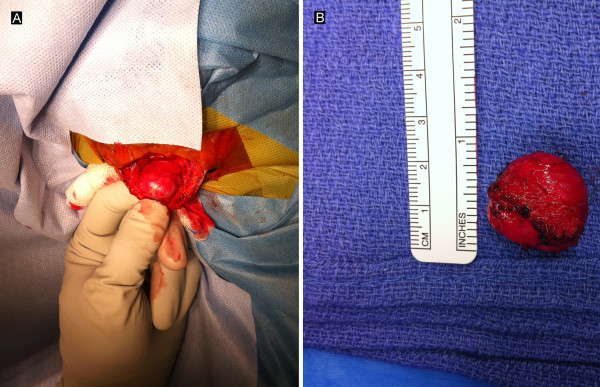
**Intraoperative photograph.** Intraoperative photograph shows (**A**) the thrombosed non-pulsatile occipital artery aneurysm and (**B**) specimen after en bloc excision.

**Figure 3:**
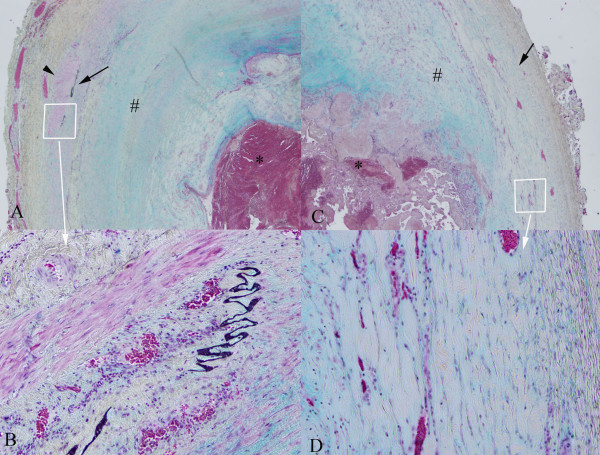
**Histopathologic examination shows a true aneurysm.** Histopathologic examination demonstrates a true aneurysm which includes all three layers of the arterial wall: the intima, media and adventitia. (**A**) and (**C**) show the artery under 20× magnification with intraluminal thrombus (*) and thickened intima (#). Residual smooth muscle fibers of the media are also seen (arrow head) in (**A**). (**C**) shows a portion of the arterial wall dilated by the aneurysm. (**B**) and (**D**) are 100× magnifications of the margin of (**A**) and (**B.**)

Gross examination of the specimen demonstrated a smooth, fluctuant, intact cyst (Figure 2). Sectioning revealed a thin, tan-white wall and red-purple, soft-to-friable contents. Microscopic examination showed a well-circumscribed thrombosed artery with fibrosis and granulation tissue (Figure 3). The lumen was filled with hemorrhage, fibrin and abundant papillary structures with fibrinous cores lined by a single layer of endothelial cells. There was no cytologic atypia, mitosis or necrosis of the endothelial cells. Movat’s pentachrome stain highlighted elastic lamina, consistent with the wall of an artery.

Our patient returned to our Pediatric Neurosurgery clinic for follow-up, and at 15 months has had no recurrence of this mass.

## Discussion

Aneurysms of the terminal branches of the external carotid artery are rare. They are generally the results of blunt, penetrating or iatrogenic trauma but can also be associated with infections. Traumatic aneurysms usually develop two to six weeks after blunt head trauma. Pseudoaneurysms are more common in the scalp and do not involve all layers of the arterial wall. True aneurysms, however, involve all three vessel layers - the intima, media and adventitia - and represent a localized or diffuse dilatation of the vessel wall.

The occipital artery has three segments. From proximal-to-distal-most, they are the digastric, suboccipital and subgaleal segments. At the level of the superior nuchal line, the suboccipital segment of the artery crosses the sagittal plane, intersecting the midpoint of the lambdoid suture on the ipsilateral side [1]. Here, the artery becomes vulnerable to blunt trauma because of its exposed position overlying the occipital bone.

There have only been 10 previous reports of traumatic and spontaneous aneurysm of the occipital artery (Table 1). Six cases of occipital artery aneurysm were identified as pseudoaneurysms during pathologic examinations. Only one previously reported case of a true occipital artery aneurysm has been reported: atherosclerotic change and hemodynamic stress to the arterial wall may have contributed to its development in a 51-year-old man [2]. Congenital vulnerabilities of the arterial wall, such as defects of the elastic membrane, may contribute to the development of a true aneurysm after minor head trauma, as may have been the case in our patient [3].

**Table 1:** Ten previously reported cases of occipital artery aneurysms, including the current case

**Author/year**	**Age (years)/sex**	**Presentation/etiology**	**Procedure**	**Pathology**	**Follow-up**
Yang *et al*., 2005 [4]	85 F	Post-traumatic. At two weeks post-injury non-tender, non-pulsatile mass noted. Patient then presented two months later with scalp bleeding from mass eroding through skin.	Direct puncture embolization	No formal pathology	Resolution of symptoms. No recurrence at six months.
Aquilina *et al*., 2005 [1]	15 M	Post-traumatic. Painful, enlarging, pulsatile mass four weeks after injury with occipital headache.	Resection	Pseudoaneurysm	Postoperative resolution of symptoms.
Tambasco *et al*., 2007 [5]	68 F	Iatrogenic after deep brain stimulation lead tunneling. Painful pulsatile mass two weeks after surgery.	Endovascular embolization	No formal pathology	Non-pulsatile immediately after embolization. Mass disappeared in one month.
Anan *et al*., 2008 [6]	81 F	Post-traumatic. Two years after injury, incidentally discovered during workup of brain metastasis.	No intervention	Pathology unknown	Stable on angiography two years after incidental discovery.
Patel *et al*., 2008 [7]	85 F	Post-traumatic. Three weeks after injury, presented with pulsatile, firm, non-tender mass.	No intervention	Pathology unknown	Mass involuted during observation period. No recurrence at one year.
John *et al*., 2009 [8]	16 M	Post-traumatic. Painful, enlarging, pulsatile mass six months after injury.	Resection	Pseudoaneurysm	Resolution of symptoms. No recurrence at one year.
Kanematsu *et al*., 2010 [9]	48 M	Spontaneous, NF-1 associated. Patient presented with painful neck swelling and bleeding after rupture of spontaneous aneurysm of occipital artery.	Endovascular coil embolization	No formal pathology	Bleeding stopped by procedure. No recurrence at 28 months.
	39 M	Spontaneous, NF-1 associated. Patient presented with painful neck after rupture of spontaneous aneurysm of occipital artery.	Endovascular coil embolization	No formal pathology	No recurrence at six months.
Kim *et al*., 2010 [2]	51 M	Spontaneous. Painless, pulsatile scalp mass in left occipital area; no history of trauma.	Resection	**True aneurysm**	Four months without radiographic evidence of recurrence.
Kim *et al*., 2010 [10]	36 M	Spontaneous. Pulsatile mass in right suboccipital region for one year with no history of trauma.	Resection	Pseudoaneurysm	Unknown. Follow-up not reported.
Present case	14 M	Post-traumatic. Non-pulsatile painless scalp mass at site of injury two months prior. Excised at four months due to persistence.	Resection	**True aneurysm**	Resolution of symptoms. No recurrence at 15 months.

*F* female, *M* male, *NF-1* neurofibromatosis type 1.

This disorder may not be readily diagnosed based on history and physical examination, especially if the scalp mass is a thrombosed, non-pulsatile vascular lesion. A high degree of clinical suspicion is required to recognize an occipital artery aneurysm or pseudoaneurysm. Differential diagnosis of the occipital scalp mass should include dermoid or epidermoid cyst, eosinophilic granuloma, hematoma, abscess, aneurysm, arteriovenous fistula, encephalocele, lymphoid hyperplasia and sinus pericranii [11]. While, in most cases, surgical resection of this lesion is a straightforward procedure with low morbidity, an issue may arise in young pediatric patients for whom blood loss is a significant concern. For this reason, physicians should take care not to miss this pathology in the pediatric population.

If clinically suspected, other diagnostic tools may be essential for correct diagnosis. Duplex ultrasound shows fusiform dilation and turbulent intraluminal arterial flow in non-thrombosed aneurysms [12]. Computed tomography angiography can provide important information on the vessel of origin, luminal morphology and relationship to adjacent osseous and soft tissue structures [13]. Conventional angiography is considered the gold standard for defining these lesions and differentiating them from arteriovenous malformations, which also present as pulsatile subcutaneous masses [14]; however, it may be less useful in cases of thrombosed aneurysms.

Although the natural history of this rare lesion is unknown, indications in the treatment of occipital artery aneurysm have included reduced risk of hemorrhage, pain relief and, in most cases, the alleviation of cosmetic disfigurement. In our case, the indication for surgery was for definitive pathologic diagnosis of the scalp mass and cosmesis. Treatment options for occipital aneurysms include simple resection, proximal ligation of the parent artery, trapping of the aneurysm, percutaneous ultrasound-guided thrombosis of the lesion, and endovascular arterial embolization or coil occlusion [7,15].

## Conclusions

Aneurysms of the occipital artery are rare, and the majority of them are pseudoaneurysms. When true aneurysms do occur, they present as painless swelling. Neurological complications are exceedingly rare. The diagnosis may be made preoperatively by careful physical examination if the clinical suspicion is high. In other instances, however, the diagnosis of an aneurysm is not made until pathologic review after surgical resection of the scalp mass. Preoperative diagnosis is especially difficult in the case of thrombosed aneurysms. Surgical resection is generally curative. Care should be taken in very young children due to a potential for blood loss during surgery.

## Consent

Written informed consent was obtained from the patient’s parents for publication of this case report and accompanying images. A copy of the written consent is available for review by the Editor-in-Chief of this journal.

## Competing interests

The authors declare that they have no competing interests.

## Authors’ contributions

VYR and AJ drafted the manuscript for important intellectual content. VYR, SWH, AMA and AJ made substantial editorial revisions to the manuscript. AJ made major contributions to conception and design. All authors read and approved the final manuscript.
